# Supplementary material for: The interactome of intact mitochondria by cross-linking mass spectrometry provides evidence for coexisting respiratory supercomplexes
Source: Mol Cell Proteomics. 2017 Dec 8;17(2):216–32. doi: 10.1074/mcp.RA117.000470 (PMC5795388; doi:10.1074/mcp.RA117.000470)
Supplement: Supplemental Data [file supp_RA117.000470_133922_0_supp_23978_fzffwf.zip › spectra_annotation/mito_spectra_annotation/384-1-10-1-8-1.pdf]

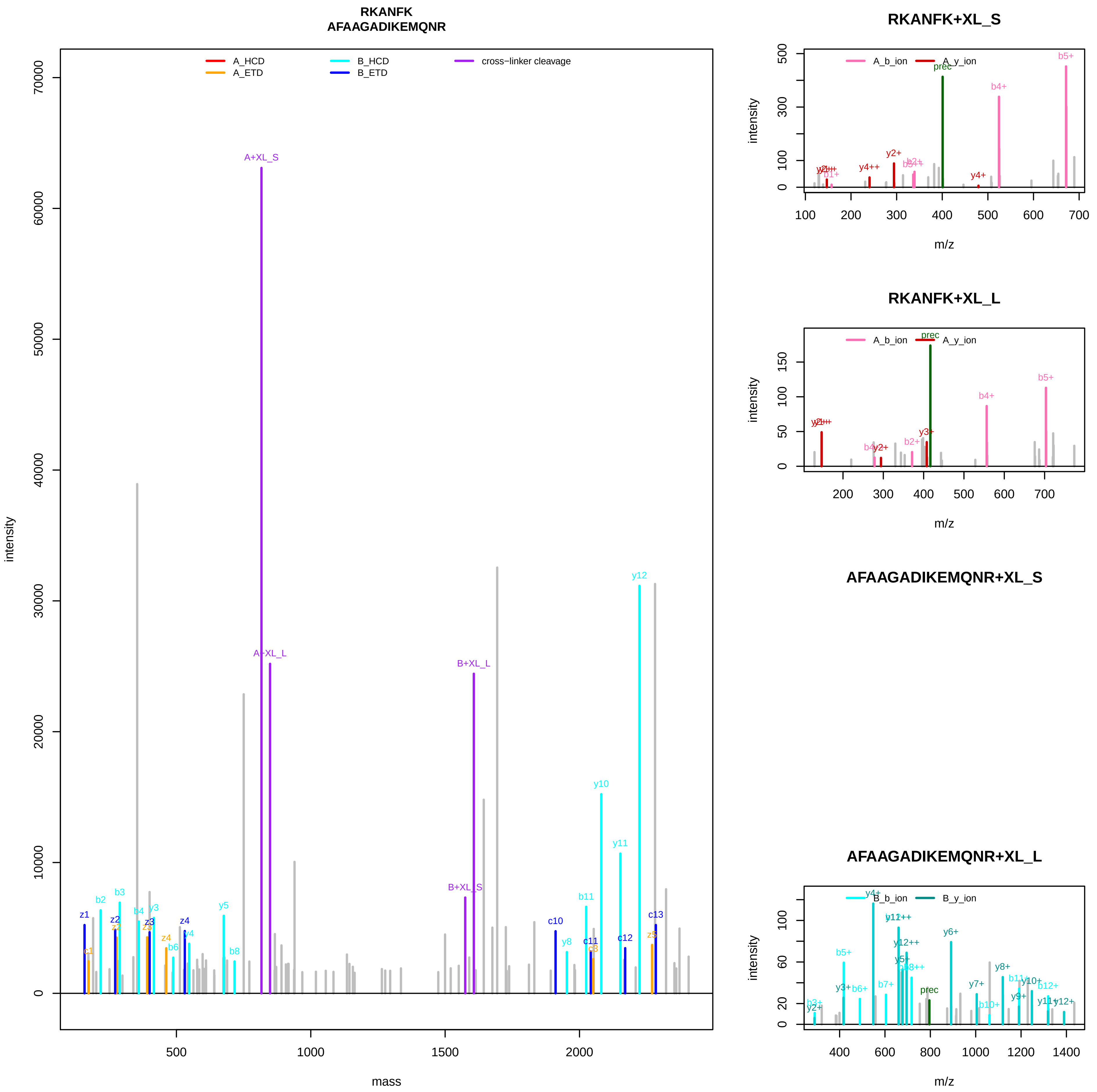
RKANFK
AFAAGADIKEMQNR
A_HCD
A_ETD
B_HCD
B_ETD
cross−linker cleavage
A+XL_S
A+XL_L
B+XL_L
B+XL_S
z1
c1
b2
z2
z2
b3
b4
z3
z3
y3
z4
b6
z4
y4
y5
b8
c10
y8
b11
c11
c3
y10
y11
y12
c12
c13
z5
intensity
mass
RKANFK+XL_S
A_b_ion
A_y_ion
prec
b5+
b4+
y2+
y4++
y4+
b1+
m/z
RKANFK+XL_L
A_b_ion
A_y_ion
prec
b5+
b4+
y2+
y3+
b2+
y2+
m/z
AFAAGADIKEMQNR+XL_S
AFAAGADIKEMQNR+XL_L
B_b_ion
B_y_ion
y4+
y12++
y12++
y6+
b5+
y5+
y3+
b6+
b7+
prec
y7+
y8+
b10+
y9+
y10+
b12+
y11+
y12+
y2+
b3+
m/z
intensity
